# Integrative Analysis of Identifying Methylation-Driven Genes Signature Predicts Prognosis in Colorectal Carcinoma

**DOI:** 10.3389/fonc.2021.629860

**Published:** 2021-06-11

**Authors:** Hao Huang, Jinming Fu, Lei Zhang, Jing Xu, Dapeng Li, Justina Ucheojor Onwuka, Ding Zhang, Liyuan Zhao, Simin Sun, Lin Zhu, Ting Zheng, Chenyang Jia, Binbin Cui, Yashuang Zhao

**Affiliations:** ^1^ Department of Epidemiology, Public Health School of Harbin Medical University, Harbin, China; ^2^ Department of Colorectal Surgery, The Third Hospital of Harbin Medical University, Harbin, China

**Keywords:** colorectal cancer, methylation-driven genes, prognostic risk model, integrative analysis, overall survival

## Abstract

**Background:**

Aberrant DNA methylation is a critical regulator of gene expression and plays a crucial role in the occurrence, progression, and prognosis of colorectal cancer (CRC). We aimed to identify methylation-driven genes by integrative epigenetic and transcriptomic analysis to predict the prognosis of CRC patients.

**Methods:**

Methylation-driven genes were selected for CRC using a MethylMix algorithm and LASSO regression screening strategy, and were further used to construct a prognostic risk-assessment model. The Cancer Genome Atlas (TCGA) database was obtained as the training set for both the screening of methylation-driven genes and the effect of genes signature on CRC prognosis. Then, the prognostic genes signature was validated in three independent expression arrays of CRC data from Gene Expression Omnibus (GEO).

**Results:**

We identified 143 methylation-driven genes, of which the combination of *BATF*, *PHYHIPL*, *RBP1*, and *PNPLA4* expression levels was screened as a better prognostic model with the best area under the curve (AUC) (AUC = 0.876). Compared with patients in the low-risk group, CRC patients in the high-risk group had significantly poorer overall survival in the training set (HR = 2.184, 95% CI: 1.404–3.396, *P* < 0.001). Similar results were observed in the validation set. Moreover, VanderWeele’s mediation analysis indicated that the effect of methylation on prognosis was mediated by the levels of their expression (HR_indirect_ = 1.473, *P* = 0.001, Proportion mediated, 69.10%).

**Conclusions:**

We identified a four-gene prognostic signature by integrative analysis and developed a risk-assessment model that is significantly associated with patients’ survival. Methylation-driven genes might be a potential prognostic signature for CRC patients.

## Introduction

Colorectal cancer (CRC) is the most common malignant tumor of the digestive system (1). Although recent advances in diagnostic and therapeutic modalities for CRC have greatly improved in survival with early colorectal carcinoma, the 5-year overall survival (OS) rates remain low in the late stage of CRC (2, 3). According to the SEER database (1973–2014, 2017 release), the 5-year survival rate for stage IV patients with metastases is only 11% (4). Nowadays the tumor-node-metastasis (TNM) staging system is identified as the gold standard to determine the prognosis of CRC patients. However, the effects and prognosis of CRC patients in the same stage using the same treatment are very different, demonstrating that there is the heterogeneity of tumor prognosis in the same stage and thus, the traditional TNM staging system fails to reflect tumor heterogeneity and assess the prognosis of CRC patients accurately (5, 6). Therefore, more effective prognostic biomarkers are needed to evaluate CRC prognosis.

DNA methylation is one of the most frequently occurring epigenetic modifications, which plays a crucial role in regulating gene expression and genome function (7). A series of studies have reported significant biomarkers for predicting the prognosis of CRC patients at different omics levels, including DNA methylation (8), microRNAs (9), gene expression (10), and proteins (11). These studies are based on single-level OMICS to consider the complicated process of tumor development (12). While the multi-OMICS may understand the biological behavior of tumors more systematically in multiple dimensions to further reveal complex molecular mechanisms in different phenotypic manifestations and discover molecular candidates with prognostic values (13). Recent studies have a trend of integrating omics to better screen potential prognostic biomarkers (14, 15). Currently, there is a driven regulation mode for selective recognition of hypermethylated or hypomethylated genes that can regulate gene expression and form specific tissue types during development (16). This mode may identify methylation-driven genes, which serve as a key indicator in the development, progression, and prognosis of tumors. At present, studies on methylation-driven genes to evaluate the prognosis of patients have been reported in the bladder (17), hepatocellular (18), and gastric cancers (19). Therefore, it is imperative to combine the profiles of DNA methylation and expression to identify CRC-related methylation-driven genes and evaluate the prognosis of CRC patients.

Here, CRC-related specific methylation-driven genes were based on the MethylMix algorithm. These genes were selected by the profiles of genome-wide DNA methylation and gene expression from The Cancer Genome Atlas (TCGA) and were validated from ArrayExpress databases. We further constructed a prognostic model to predict the overall survival (OS) of CRC patients in TCGA datasets and validated this model by Gene Expression Omnibus (GEO) datasets. The time-dependent receiver operating characteristic (ROC) curves and nomograms were utilized to estimate the capability of prediction for the prognostic model in two datasets.

## Materials and Methods

### Study Population and Data Preprocessing

All the subjects used in this study were obtained from publicly available databases, including TCGA, GEO, and ArrayExpress databases. Methylation-driven genes for CRC were identified by the profiles of DNA methylation and gene expression from TCGA (N = 431), including 386 CRC tissues and corresponding 45 adjacent non-tumor tissue samples. Then these candidate genes were validated further from ArrayExpress databases (N = 214) where contain 214 CRC tissue samples. A prognostic risk-assessment model was developed based on TCGA datasets (N = 367) and was validated the model by Gene Expression Omnibus (GEO) datasets (N = 355) of three-independent gene expression arrays [GSE17536 (N = 177), GSE17537 (N = 55), and GSE72970 (N = 123)], where the CRC clinical information included sex, age, TNM stage, and survival.

Level 3 methylation data were obtained from the TCGA Methylation 450k Bead chip by the function of the DownloadMethylationData in a TCGA-Assembler 2 Bioconductor package (18, 20). According to the function of the CalculateSingleValueMethylationData, the average value of all CpG sites in the promoter region between the transcription start site (TSS) 200 and TSS 1,500 bps was calculated. Meanwhile, RNA-seq expression data were also collected from TCGA database. The RNA-Seq data were normalized by function ProcessRNASeqData.

### Identification and Validation of Methylation-Driven Genes for CRC

MethylMix is an R package using the analysis of the correlation between methylation level and gene expression level (21). According to the Bioconductor package MethylMix, we integrated DNA methylation data of the tumor tissue samples and normal tissue samples, and gene expression data of CRC tissue samples in TCGA datasets to screen most likely specific driven genes for CRC. The highly correlated genes were selected for further analyses. We compared the DNA methylation status in tumor versus normal patients by Wilcoxon rank-sum test. Absolute log fold change (FC) ≥0, correlation coefficient (Cor) < −0.5 and adjusted *P* < 0.05 were used as screening conditions. Finally, we screened out 143 methylation-driven genes for further analyses according to the requirements of the MethylMix algorithm. To further narrow the predictors, a least absolute shrinkage and selection operator (LASSO) regression was used to narrow the range of methylation-driven genes. A strong correlation often exists between the variables, indicating that high dimensionality and collinearity. And this LASSO model method could decrease the characteristic dimension. Then, a multivariable Cox regression model to select driven genes that were most closely associated with survival was constructed and six methylation-driven genes were retained (22, 23).

Moreover, a total of 214 CRC patients contained both DNA methylation and expression data were collected from patients for surgery at the Royal Brisbane and Women’s Hospital in Brisbane, Australia, a consecutive manner between 2009 and 2012 (24). We analyzed these six methylation-driven genes whose correlation between the methylation levels of promoter probes and those gene expressions to further validate whether are the candidate methylation-driven genes. The correlation between methylation level in the promoter region and their corresponding gene expression level was calculated by Pearson’s rank. The data have been stored at EMBL-EBI (https://www.ebi.ac.uk/arrayexpress/) from the ArrayExpress database. The accession numbers are E-MTAB-7036 (methylation) and E-MTAB-8148 (expression).

### Construction and Validation of a Prognostic Risk-Assessment Model

To better assess the prognostic predictive power of those methylation-driven genes, we construct a prognostic risk-score model by multivariable Cox analysis:

$$Risk\;score\;(RS)=\sum_{i=1}^{N}(Exp\times Coef),$$

In which, N represents the number of methylation-driven genes; Exp is the expression level of every driven gene, and Coef is the coefficient of multivariable Cox regression analysis in the model. Risk score (RS) is a multimode weighted sum of the prognostic risk value of each sample. Six methylation-driven genes could combine 2^n^−1 = 63 signatures, therefore, every CRC patient has 63 prognostic risk scores. In the training set, the hazard ratios (HR) and the area under curves (AUCs) values from the prognostic score of the 63 signatures were analyzed. We constructed the best prognostic risk model by comparing each AUC value in 63 signatures.

To validate the predictive capability of the best risk-assessment model, we obtained three gene expression arrays of human CRC datasets [GSE17536 (N = 177), GSE17537 (N = 55), and GSE72970 (N = 123)] from the Gene Expression Omnibus (GEO) (https://www.ncbi.nlm.nih.gov/geo/), serving as a validation cohort (N = 355) (25–27). To minimize batch effects from different microarray platforms, samples in three different datasets were selected from the same chip platform (Affymetrix Human Genome U133 Plus 2.0 Array) and normalized with by Bioconductor package Sva (28, 29).

### Gene Set Enrichment Analysis (GSEA)

To explore the potential biological function and promising signaling pathways correlated with the methylation of driven genes, GSEA was conducted to analyze the biological function of four genes using the Java GSEA v4.0.1 software (http://software.broadinstitute.org/gsea/datasets.jsp). The files of ontology gene sets were collected from the Gene Ontology (GO) (c5.all.v7.1.symbols) and the Kyoto Encyclopedia of Genes and Genomes (KEGG) (c2.cp.kegg.v7.1.symbols) databases. The screening conditions of significant pathways and biological functions were the absolute value of normalized enrichment score (NES) >1, *P*-value <0.05, and false discovery rate (FDR) q value <0.05.

### Statistical Analysis

The median cut-off value divided CRC patients into high-risk and low-risk groups. The analysis of time-dependent ROC curves and Kaplan-Meier survival analysis were utilized to compare the survival rates at different follow-up time points and the difference of the OS between the two groups for CRC patients. Then, univariable and multivariable Cox regression analyses were performed to illustrate whether the methylation signature model is serving as an independent indicator. Before conducting multivariable Cox regression models, we successfully estimated the assumption by the equal-proportional hazards assumption. Moreover, in order to evaluate further the survival probability of individual patient’s outcome events, the clinical factors (age, gender, and TNM staging) and risk score of genes signature were used to build the nomogram by utilizing the rms and the Hmisc packages in R. In the nomogram, each patient had a score for predicting each survival probability, and a higher number of total points represented a worse outcome for the patient. Calibration curves were calculated to estimate the efficiency of the nomogram. VanderWeele’s mediation analysis was utilized to explore whether the effect of the methylation signature on prognosis is affected by their mRNA expression (30). The total effect of methylation on prognosis (HR_Total_) was split into two effects, including the direct effect (HR_Direct_) which represents the direct effect of the methylation on prognosis, and the indirect effect (HR_Indirect_) that indicates the prognostic effect of methylation mediated through gene expression. All analyses were performed with the R Statistical Program (version 3.6.1). *P*-value <0.05 were considered statistically significant.

## Results

### Clinical Characteristics of the Patients

The clinical information of CRC patients contained a training cohort (N = 367) that was extracted from the TCGA database and a validation cohort (N = 355) that was obtained from GEO datasets (GSE17536, GSE17537, and GSE72970). The patients’ characteristics are summarized in **Table 1**.

**Table 1:** Summary of patient demographics and clinical characteristics.

Characteristics	Groups	Patients					
		Total (N = 722)		Training set (N = 367)		Testing set (N = 355)	
		No.	%	No.	%	No.	%
Age at diagnosis							
	Median	65.3		64.4		63.7	
	Range	21.0–97.0		31.0–90.0		21.0–94.0	
	<65 years	354	49.0	172	46.9	182	51.3
	≥65 years	368	51.0	195	53.1	173	48.7
Gender							
	Male	394	54.6	199	54.2	195	54.9
	Female	328	45.4	168	45.8	160	45.1
TNM stage							
	I	86	11.9	55	15.0	31	8.7
	II	216	29.9	141	38.4	75	21.1
	III	208	28.8	117	31.9	91	25.6
	IV	212	29.4	54	14.7	158	44.5
Vital status							
	Living	458	63.4	287	78.2	171	48.2
	Dead	264	36.6	80	21.8	184	51.8

### Identification and Validation of CRC Methylation−Driven Genes

**Figure 1:**
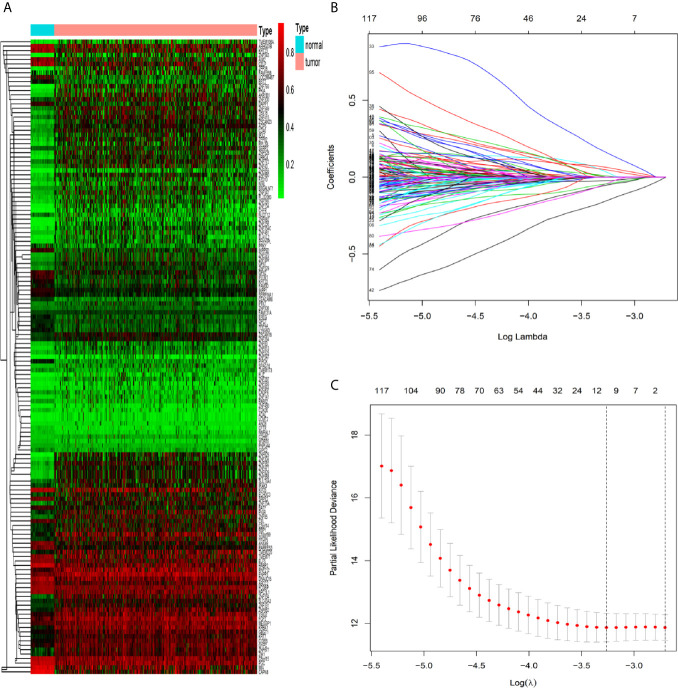
Identification of methylation-driven genes in CRC patients. **(A)** Heat map of 143 CRC-related methylation-driven genes. The color change from green to red illustrates a trend from hypomethylation to hypermethylation. |log FC|≥0, adjusted *P* < 0.05, and Cor <−0.5. CRC, colorectal cancer; FC, fold change. **(B)** Selection of driven genes in the LASSO model. **(C)** Tuning parameter (λ) selection in the LASSO model used cross-validation *via* the maximum criteria. The dotted vertical lines were drawn at the optimal values using the maximum criteria and the one standard error of the maximum criteria.

By the MethylMix algorithm, we identified 143 methylation-driven genes that were transcriptionally regulated with methylation status. The process of determining and analyzing methylation-driven genes signature is displayed in **Supplementary Figure S1**. These genes are summarized in **Figure 1A** and **Supplementary Table S1**. After screening out these 143 methylation-driven genes, we included these genes in the LASSO model. We found that when the λ value is 0.038, the cross-validation error coefficient of the model is lowest, and the corresponding genes are ten (*ANXA9*, *BATF*, *PHYHIPL*, *RBP1*, *PNPLA4*, *FCGBP*, *GIPC2*, *FGC2*, *FAM131A*, and *SERPINA1*) (**Figures 1B, C**). Then, 10 genes obtained by the LASSO regression model were incorporated into the multivariable Cox model. And finally obtained six methylation-driven genes (*ANXA9*, *BATF*, *PHYHIPL*, *RBP1*, *PNPLA4*, and *SERPINA1*) (**Supplementary Table S2**). We further validated the correlation between methylation level of probes in the promoter region and corresponding gene expression level in a total of 214 patients from the ArrayExpress database. Due to the partially missing in the methylation 450K bead chip data, we validated only four methylation-driven genes (*ANXA9*, *BATF*, *RBP1*, and *SERPINA1*). However, the stable results of candidate genes were similar to training sets (**Supplementary Figure S2**).

### Construction and Validation of the Prognostic Risk-Assessment Model in the Training and Testing Sets

**Figure 2:**
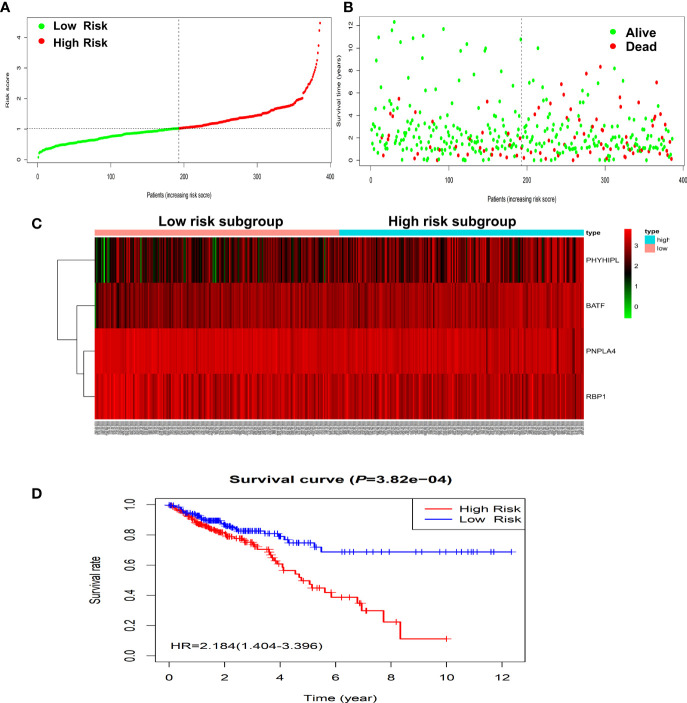
Construction of four-gene risk score model in the TCGA dataset. **(A)** Distribution of risk scores in the high-risk and low-risk groups. **(B)** Survival overview in two high-risk and low-risk groups. **(C)** Heatmap of the four-gene expression profiles corresponding risk scores in the high-risk and low-risk groups in the TCGA database. **(D)** Comparison of OS between the high-risk and low-risk groups. OS, overall survival.

According to the risk score of the prognostic model in the training set, these six methylation-driven genes have 2^6^−1 = 63 possible combinations and relevant prognostic risk scores. By calculating AUC values of 63 signatures, we found that the expression signature consisted of *BATF*, *PHYHIPL*, *PNPLA4*, and *RBP1* was served as a better prognostic signature (**Supplementary Table S3**). The prognostic risk score of these combined four genes was determined as follows: Risk score = (0.253 × expression level of *BATF*) + (0.147 × expression level of *PHYHIPL*) + (−0.183 × expression level of *PNPLA4*) + (−0.172 × expression level of *RBP1*) (**Table 2**). The AUC value of four methylation-driven genes signature was 0.876, demonstrating a better capability of prediction with the 9-year OS of CRC patients. The Kaplan-Meier survival analysis demonstrated that CRC patients in the high-risk group had poorer survival than those in the low-risk group (HR = 2.184, 95% CI: 1.404–3.396, *P* < 0.001) (**Figure 2**). Moreover, we further analyzed the difference of expression levels of four genes in tumor and normal tissues and found that the expression level of *PHYHIPL* (*P* = 0.002) in CRC tissues is lower than that of normal tissue. While the expression level of *BATF* in normal tissue is lower than that of CRC tissue (*P* = 0.002). However, the expression levels of *PNPLA4* and *RBP1* are not significantly different between CRC tissue and normal tissue (**Supplementary Figure S3**).

**Table 2:** Identified four methylation-driven genes in the prognostic signature and their multivariable Cox associated with prognosis.

Gene symbol	Coefficient^a^	HR	HR (95% Low)	HR (95% High)	*P*-value^a^
*BATF*	0.253	1.288	1.088	1.526	0.003
*PHYHIPL*	0.147	1.158	1.046	1.282	0.005
*PNPLA4*	−0.183	0.833	0.691	1.003	0.053
*RBP1*	−0.172	0.842	0.732	0.968	0.015

a Derived from the multivariable Cox regression analysis in the training set.

To validate the predictive capability of the expression prognostic genes signature, the same prognostic model was used to calculate the risk scores of a total of 355 CRC patients in the independent testing set of the GEO database. The Kaplan-Meier survival analysis showed CRC patients in the high-risk group had significantly poorer survival than those in the low-risk group (HR = 1.963, 95% CI: 1.456–2.647, *P* < 0.001) (**Supplementary Figure S4**). These results were similar to those in the training set.

**Figure 3:**
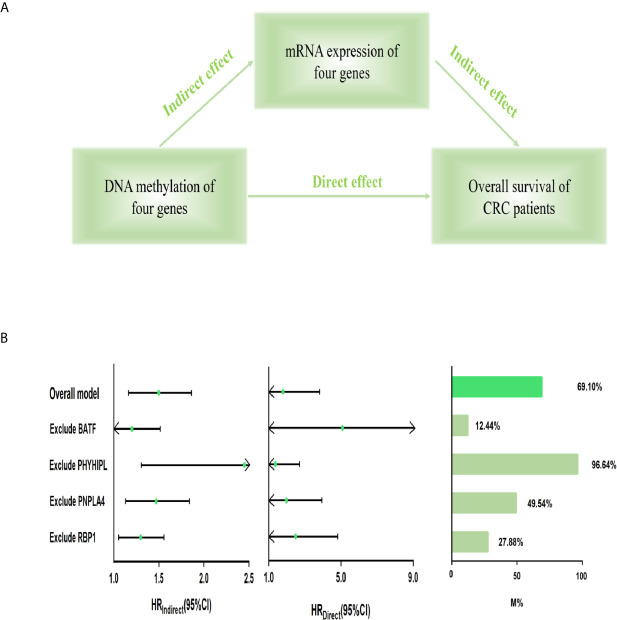
Mediation analysis for methylation-driven prognostic signature through mRNA expression. **(A)** Diagram of a mediation model. **(B)** The risk score of four methylation-driven genes’ methylation level was considered as “exposure” (score_methylation_); the mediator was the linear combination of the corresponding four genes’ expression level (score_expression_) (Overall model). Total prognostic effect in the hazard ratio (HR) was described as direct effect (HR_direct_), indirect effect (HR_indirect_), corresponding 95% CI, and the proportion of effect mediated (M%). Furthermore, sensitivity analyses were performed by excluding each gene, respectively, which retained statistical significance for the mediation effect. CI, confidence interval.

Furthermore, we built the mediation model underlying the mediation pathway of methylation, mRNA expression, and OS by VanderWeele’s mediation analysis (**Figure 3A**). The effect of the methylation signature of combined four genes on prognosis was mostly mediated by their corresponding mRNA expression (HR_indirect_ = 1.473, 95% CI: 1.165–1.862, *P* = 0.001, Proportion mediated, 69.10%). After excluding the methylation and expression of each gene, the result of sensitivity analysis retained statistically significant in the indirect effect (**Figure 3B**).

### Assessment of the Predictive Performance of the Expression Prognostic Model by Time-Dependent ROC Curves and the Nomogram  

**Figure 4:**
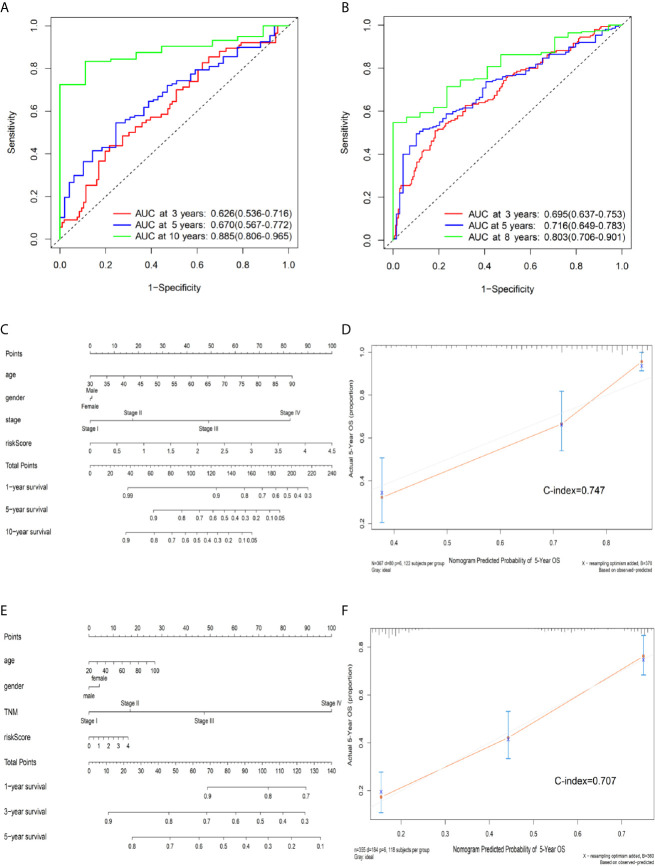
Predictive OS performance of the signature using time-dependent ROC analysis and the nomogram in training and validation sets. **(A)** Time-dependent ROC curves analysis for the 3-, 5-, and 10-year OS prediction by signature in the training set. **(B)** Time-dependent ROC curves analysis for the 3-, 5-, and 8-year OS prediction by signature in the testing set. **(C)** Nomogram to predict the 1-, 5-, and 10-year OS of CRC patients in the training set. **(D)** Calibration curves of 5-year OS nomogram model in the training set. **(E)** Nomogram to predict the 1-, 3-, and 5-year OS of CRC patients in the testing set. **(F)** Calibration curves of 5-year OS nomogram model in the testing set. The gray line represents the ideal predictive model, and the red line represents the observed model.

According to a time-dependent ROC curves analysis, in the training set, we observed that their AUC values were 0.626 at 3 years, 0.670 at 5 years, and 0.885 at 10 years, respectively (**Figure 4A**). We further observed AUC values in the testing set, with 3-, 5-, and 8-year were 0.695, 0.716, and 0.803, respectively (**Figure 4B**). Then, we investigated whether the risk score of genes signature was used as an independent indictor for CRC patients by univariable and multivariable Cox analyses, and found that the prognostic score was an independent prognostic factor in the training set (high-risk group *vs* low-risk group, HR = 2.221, 95% CI: 1.382–3.571, *P* = 0.001). However, the result in the testing set was a little bit low (high-risk group *vs* low-risk group, HR = 1.436, 95% CI: 1.051–1.962, *P* = 0.023) (**Table 3**).

**Table 3:** Univariable and multivariable Cox regression analyses of the four methylation-driven genes signature and survival of CRC patients in the training and testing sets.

Variables	Training set (N = 367)				Testing set (N = 355)			
	95% CI				95% CI			
	HR	Lower	Upper	*P*	HR	Lower	Upper	*P*
**Univariable analysis**								
Age								
≥65 years *vs <*65 years	2.170	1.328	3.547	0.002	0.938	0.702	1.253	0.664
Sex								
Male *vs* female	1.449	0.923	2.274	0.107	0.958	0.717	1.282	0.774
TNM stage								
III+IV *vs* I + II	2.765	1.741	4.391	0.000	4.251	2.742	6.591	0.000
**Four genes signature**								
High risk *vs* low risk	2.351	1.472	3.755	0.000	1.963	1.456	2.647	0.000
**Multivariable analysis**								
Age								
≥65 years *vs*<65 years	2.355	1.421	3.903	0.001	1.270	0.942	1.712	0.117
Sex								
Male *vs* female	1.123	0.712	1.771	0.618	0.942	0.702	1.264	0.690
**TNM stage**								
III+IV *vs* I + II	3.291	2.049	5.286	0.000	3.967	2.508	6.274	0.000
**Four genes signature**								
High risk *vs* low risk	2.221	1.382	3.571	0.001	1.436	1.051	1.962	0.023

We further built a nomogram, including the risk score of signature and clinical factors (age, gender, and TNM stage). The nomogram served as an individual’s prognostic predictor to predict the probability of overall survival with 1-, 5-, and 10-year for CRC patients (**Figure 4C**). Moreover, in the training set, calibration plots demonstrated that the nomogram had similar predictive performance compared with an ideal model in predicting the 5-year OS for CRC patients (**Figure 4D**). Similar results were observed in the testing set (**Figures 4E, F**) (Concordance-index: 0.747 in the training set and 0.707 in the testing set). Additionally, compared with the TNM staging system, the nomogram had a higher C-index in predicting the OS for CRC patients in the training and testing sets (**Supplementary Table S4**).

### Subgroup Analyses of the Prognostic Performance of the Methylation-Driven Genes Signature

To determine whether our model was highly applicable and precisely predict the OS of CRC patients, we performed subgroup analyses based on different clinical characteristics (age, gender, and TNM stage). The prognostic effect of the genes signature in different age groups, female groups, TNM stage groups revealed that CRC patients in the high-risk group had significantly poorer survival than those in the low-risk group (*P* < 0.001). However, in the male, similar results could not be observed in the training set (**Supplementary Figure S5**). Similar results were also observed in the testing set (**Supplementary Figure S6**).

### Comparison of Prognostic Risk Model With Other Prognostic Biomarkers in CRC

The ROC curves analysis for other prognostic biomarkers was analyzed just as our expression prognostic risk model, the results indicated that the AUC value of our four-gene signature was better than that of other known prognostic biomarkers (AUC = 0.794). The AUC values of these biomarkers are summarized in **Supplementary Figure S7** and **Supplementary Table S5**. These results revealed that our genes signature had better predictive performance in predicting the long-term OS of CRC patients.

### Functional Enrichment Analysis of Four Methylation-Driven Genes

We further explored the biological functions of the four genes by GSEA 4.0.1 software and found that the expression level of *BATF* may be related to the “regulation of viral process” and “non-small cell lung cancer.” The expression level of *PHYHIPL* may be related to the function of “blastocyst growth” and “WNT signaling pathway.” However, the FDR value is more than 0.25, there may be false-positive results. Moreover, we found that the expression level of *PNPLA4* may be related to the function of “peroxisome” in both GO and KEGG functional enrichment. The expression level of *RBP1* may be related to the “morphogenesis of a polarized epithelium” and the “WNT signaling pathway.” However, the FDR value is 1.000, there may be false-positive results (**Supplementary Figure S8**).

## Discussion

Because CRC patients with the same pathological staging often differ in survival, a new prognostic assessment model is required to indicate biological heterogeneity, appropriately guide clinical assessment and intervention, and individualize treatment (6). Previous studies have indicated that DNA methylation, an epigenetic modification, regulates gene expression in the development and progression of cancer (31). Moreover, the comprehensive analysis of DNA methylation and gene expression data can better analyze the regulatory function of methylation and effectively predict the prognosis of tumor patients (32). Therefore, methylation-driven genes may be identified as potential prognostic biomarkers with involvement in pathogenesis (17, 33). Besides, the development and progression of tumors involve the process of a complex regulatory network. Compared with a single biomarker, integrating multiple biomarkers into a combined model could better assess the prognostic value (34). We construct a prognostic model based on four methylation-driven genes and provide a comprehensive prospect for both basic research and clinical applications of methylation-driven genes.

In this study, we used different statistical analyses and the LASSO penalized model obtaining 143 methylation-driven genes. Four out of them (*BATF*, *PHYHIPL*, *PNPLA4*, and *RBP1*) were identified as genes associated with CRC prognosis, which were selected to develop a prognostic score model and validated the model in external testing set. The results showed that the prognostic score was significantly associated with the OS of CRC patients, demonstrating that CRC patients in the high-risk group have significantly poorer survival than those in the low-risk group. The AUC value based on genes signature was 0.874 in predicting the 9-year of OS for CRC patients in the training set. We further revealed that the risk score of prognostic signature could serve as an independent indictor of patient survival without the effect of age, gender, and TNM stage. Besides, the nomogram was generated to predict the survival probability of individual patients’ models, thus evaluating the probability of outcome events. The calibration plots indicated that the predicted survival was close to the actual survival status (C-index: 0.747). These results revealed the obvious predictive capability of genes signature on the prognosis of CRC patients. Moreover, in the stratified analysis, our prognostic model performed well stability for predicting the survival of CRC patients in different age, female, and TNM stage groups in the training and testing sets. However, the males’ group in the training set could not distinguish between low- and high-risk groups. Since this is the first study of methylation-driven genes for CRC, large sample sizes may be necessary to further analyze in the future. Additionally, a comparison of our prognostic signature with other prognostic biomarkers revealed that it had a higher predictive performance with OS of CRC patients.

After a series of analyses, our study provides four prognostic genes. Among these genes, three (*BATF*, *PHYHIPL*, and *RBP1*) have been reported as cancer-associated genes. *BATF*, a transcription factor, belongs to a highly conserved member of activator protein 1 (AP-1) and a family of the basic leucine zipper ATF-like transcription factor (*BATF*) (35). A series of studies suggest that *BATF* may influence the development of different types of cancer, including non-small cell lung cancer (NSCLC), lymphoma, and multiple myeloma (36, 37). Such as, *BATF* might active NSCLC cell proliferation and apoptosis in *BATF*-silenced A549 cells (38). In addition, *BATF* is a gene that inhibits T cell function, inhibitory receptors can cause T cell exhaustion by upregulating *BATF* (39). Recently a study has found that increased expression of *BATF*, a significant positive correlation that existed with *PDCD1* expression, may suppress CD8^+^ T function and affect the development of colorectal cancer (40). Phytanoyl-CoA 2-hydroxylase-interacting protein-like gene (*PHYHIPL*), a protein-encoding gene, may correlate with the prostatic small cell carcinoma (41). Not much is known about the function of *PHYHIPL* now. Previous findings from TCGA database reported that the downregulation of *PHYHIPL* is associated with poor OS, demonstrating that this gene is involved in the development of Glioblastoma multiforme (GBM) (42). *RBP1* (Retinol Binding Protein 1), is also named Cellular Retinol Binding Protein 1 (*CRBP1*) and is located in the cytogenetic region 3q23 (43). *RBP1* is considered a chaperone-like molecule to regulate the phase of retinol signaling and affect the proliferation and differentiation of epithelial cells (44). Recent studies have found that the expression of *RBP1* has been reported in many tumor cells, including breast carcinoma (45), lung adenocarcinoma (46), tongue squamous cell carcinoma (47), and cervical cancer (48). Recent studies suggest that *RBP1* hypermethylation and low expression level are associated with a poor prognosis in various cancer. For example, in EBV-associated gastric carcinoma, hypermethylation of *RBP1* in the promoter region, correlated with the upregulation of *RBP1*, which demonstrated that patients with CpG island methylator phenotype-high (CIMP-H) have poorer survival than those with CIMP-low in gastric carcinoma (49). *PNPLA4* (Patatin Like Phospholipase Domain Containing 4) belongs to a member of the patatin-like family of phospholipases, which may be involved in adipocyte triglyceride homeostasis of HeLa cells (50). Although the function of this gene is still not well known, we observed a significant negative correlation between methylation and expression level of *PNPLA4*. Therefore, *PNPLA4* may indicate a novel CRC biomarker, and further experiments are required to validate this finding.

To the best of our knowledge, this is the first predictive risk model of CRC based on methylation-driven genes. These four genes have not been previously reported on the underlying mechanism of them and studied as a prognostic biomarker in CRC patients. Our study provides a foundation for further exploration into the functions of the four genes. Other strengths include that, compared with previous studies based on methylation-driven genes in other cancers, our study firstly utilized different testing sets to separately validate methylation-driven genes and prognostic models from multi-public datasets. Additionally, we acknowledge several possible limitations to the present study. Firstly, the development and evaluation of this prognostic model were based on publicly available datasets. To further confirm this model, large sample sizes, multicenter, and prospective clinical cohorts may be necessary for the future. Secondly, studies are needed to further verify the biological mechanisms behind the values of these genes for CRC. Regardless, our results showed a significantly consistent association of the signature with OS in different datasets, demonstrating that it serves as a potential prognostic biomarker for CRC.

In summary, we identified 143 methylation-driven genes by integrative analysis of both methylation and expression profiles and selected four of them (*BATF*, *PHYHIPL*, *RBP1*, and *PNPLA4*) to construct a prognostic risk model. This study reveals that a four-gene methylation-driven prognostic signature accurately predicts the OS of CRC patients and could be a promising marker for improving the clinical prognostic evaluation of CRC patients. DNA methylation-driven genes may be a potentially useful novel biomarker for predicting CRC prognosis.

## Data Availability Statement

The original contributions presented in the study are included in the article/**Supplementary Material**. Further inquiries can be directed to the corresponding authors.

## Author Contributions

HH and JF performed research and drafted the manuscript. LZhang, JX, and DL collected the data, analyzed the data. TZ, CJ, and JO re-analysis results and interpretation. DZ, LYZ, and SS performed the figures, edited the data. YZ, BC, and LZhu revised the manuscript. All authors contributed to the article and approved the submitted version.

## Conflict of Interest

The authors declare that the research was conducted in the absence of any commercial or financial relationships that could be construed as a potential conflict of interest.

## Abbreviations

AUC, Area Under the Curve; CIMP, CpG Island Methylator Phenotype; CRC, Colorectal Cancer; C-index, Concordance Index; FC, Fold Change; GEO, Gene Expression Omnibus; GBM, Glioblastoma Multiforme; LASSO, Least Absolute Shrinkage and Selection Operator; NSCLC, Non-Small Cell Lung Cancer; ROC, Receiver Operating Characteristic; RS, Risk Score; TCGA, The Cancer Genome Atlas; OS, Overall Survival.
