# Supplementary material for: Integrative Analysis of Identifying Methylation-Driven Genes Signature Predicts Prognosis in Colorectal Carcinoma
Source: Front Oncol. 2021 Jun 11;11:629860. doi: 10.3389/fonc.2021.629860 (PMC8231008; doi:10.3389/fonc.2021.629860)
Supplement: Supplementary file 1 [file DataSheet_1.pdf]

Supplementary Material for

Integrative analysis of identifying methylation-driven genes signature predicts prognosis in colorectal carcinoma

1 List of Supplementary Material

Ⅰ Supplementary Figures

Supplementary Figure S1: Flowchart of the research.

Supplementary Figure S2: Correlation between DNA methylation and gene expression of *ANXA9*, *BATF*, *RBP1*, and *SERPINA1* in the training dataset and validation dataset.

Supplementary Figure S3: The difference of expression levels of four methylation-driven genes between tumor tissue and normal tissue.

Supplementary Figure S4: Validation of four-gene risk score model in the GEO dataset.

Supplementary Figure S5: Kaplan–Meier and ROC analyses of CRC patients based on different age, gender, and TNM staging subgroups in the training set.

Supplementary Figure S6: Kaplan–Meier and ROC analyses of CRC patients based on different age, gender, and TNM staging subgroups in the testing set.

Supplementary Figure S7: Comparison of ROC curves between the signature and other known biomarkers in predicting 8-year OS of CRC patients.

Supplementary Figure S8: Gene set enrichment analysis of candidate four methylation-driven genes.

Ⅱ Supplementary Tables

Supplementary Table S1: 143 epigenetic drivers were identified by MethylMix.

Supplementary Table S2: Identificaion of six methylation-driven genes by multivariable Cox regression analysis.

Supplementary Table S3: Compared the overall survival prediction and AUC values of 63 different combined models in the training set.

Supplementary Table S4: The C-index of TNM staging system and nomograms.

Supplementary Table S5: Compared the prognostic effect of four methylation-driven genes signature with other partially known biomarkers.

I Supplementary Figures

Supplementary Figure Legends

Supplementary Figure S1. Flowchart of the research. Identifying and validating methylation-driven genes, developing the risk score model and analyzing the efficiency of the signature to predict prognostic outcomes. TCGA, The Cancer Genome Atlas; ROC, receiver operating characteristic; AUC, area under the curve; GEO, The Gene Expression Omnibus

Supplementary Figure S2. Correlation between DNA methylation and gene expression of *ANXA9, BATF, RBP1,* and *SERPINA1* in the training dataset and validation dataset. *ANXA9*, Annexin A family member 9; *BATF*, Basic Leucine Zipper ATF-Like Transcription Factor; *RBP1*, Retinol Binding Protein 1; *SERPINA1*, Serpin family A member 1. The vertical axis and the horizontal axis denote the mRNA level and DNA methylation level, respectively

Supplementary Figure S3. The difference of expression levels of four methylation-driven genes between tumor tissue and normal tissue.

Supplementary Figure S4. Validation of four-gene risk score model in the GEO dataset. (A) Distribution of risk scores in the high-risk and low-risk subgroups. **(B)** Survival overview in two high-risk and low-risk groups. **(C)** Heatmap of the four-gene expression profiles corresponding risk scores in the high-risk and low-risk subgroups in the GEO database. **(D)** Comparison of OS between the high-risk score and low-risk score groups. OS, overall survival

Supplementary Figure S5. Kaplan–Meier and ROC analyses of CRC patients based on different age, gender, and TNM staging subgroups in the training set. Kaplan–Meier analysis was performed to estimate the differences in OS between the low-risk and high-risk patients. ROC curves of the methylation-driven gene signature were used to demonstrate the sensitivity and specificity in predicting the OS of CRC patients. OS, overall survival; ROC, receiver operating characteristic; CRC, colorectal cancer

Supplementary Figure S6. Kaplan–Meier and ROC analyses of CRC patients based on different age, gender, and TNM staging subgroups in the testing set. Kaplan–Meier analysis was performed to estimate the differences in OS between the low-risk and high-risk patients. ROC curves of the methylation-driven gene signature were used to demonstrate the sensitivity and specificity in predicting the OS of CRC patients. OS, overall survival; ROC, receiver operating characteristic; CRC, colorectal cancer

Supplementary Figure S7. Comparison of ROC curves between the signature and other known biomarkers in predicting 8-year OS of CRC patients. OS, overall survival; ROC, receiver operating characteristic; CRC, colorectal cancer

Supplementary Figure S8. Gene set enrichment analysis of candidate four methylation-driven genes. (A, B) GO (biological process) and KEGG pathway of *BATF*. (C, D) GO (biological process) and KEGG pathway of *PHYHIPL*. (E, F) GO (biological process) and KEGG pathway of *PNPLA4*. (G, H) GO (biological process) and KEGG pathway of *RBP1*. KEGG, Kyoto Encyclopedia of Genes and Genomes; GO, Gene Ontology; NES, normalized enrichment score; FDR, false discovery rate.

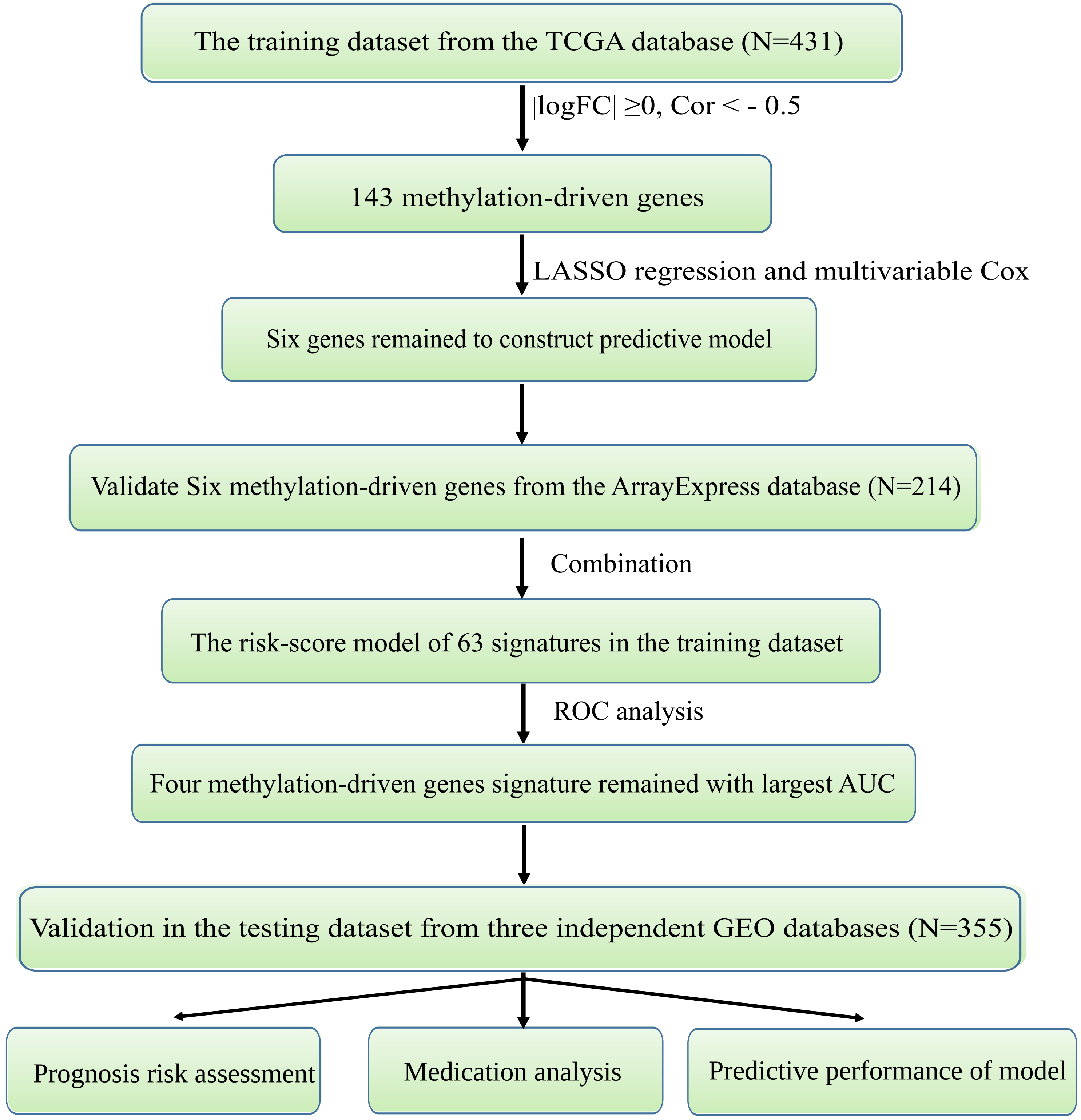

Supplementary Figure S1

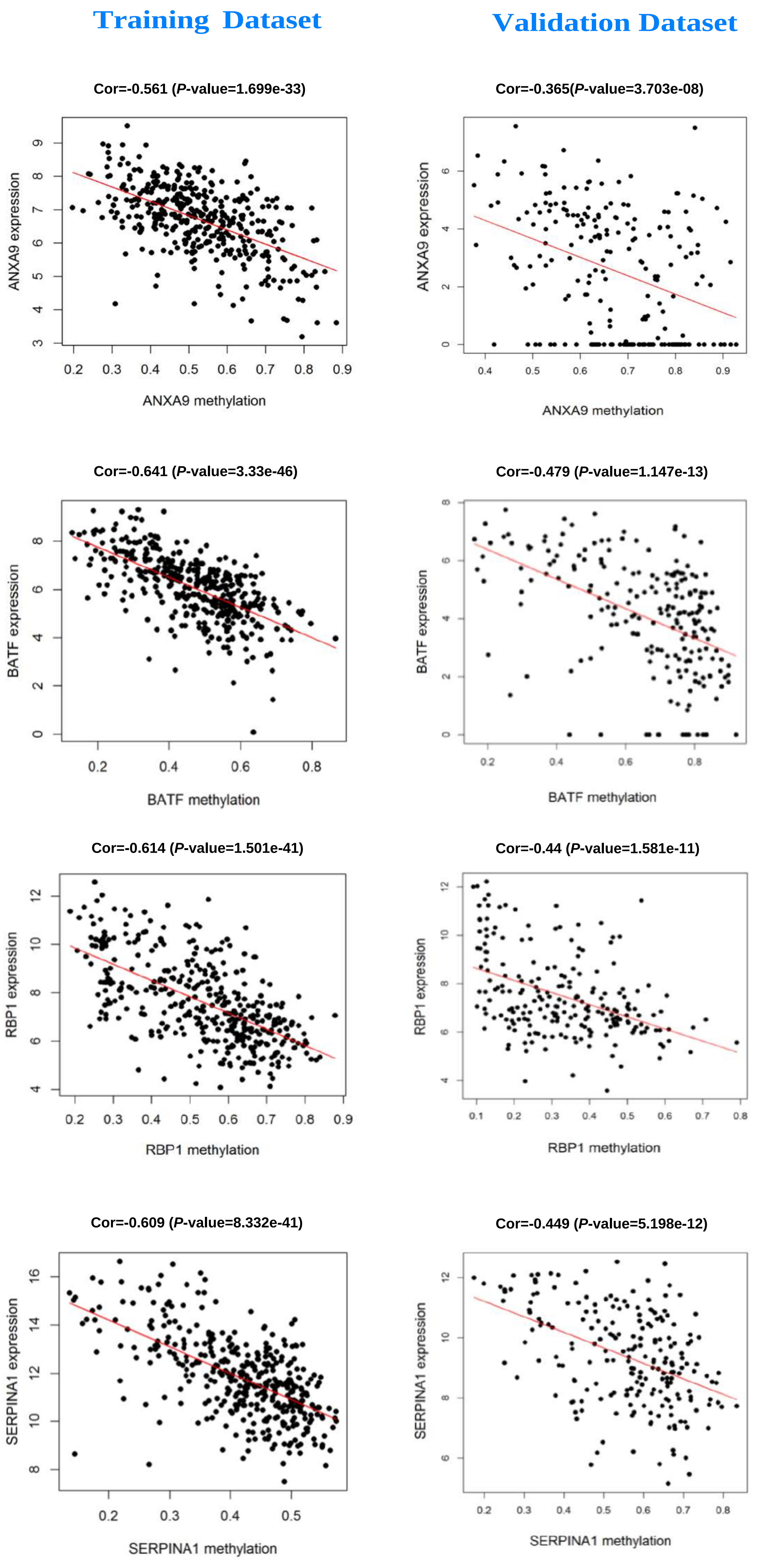

Supplementary Figure S2

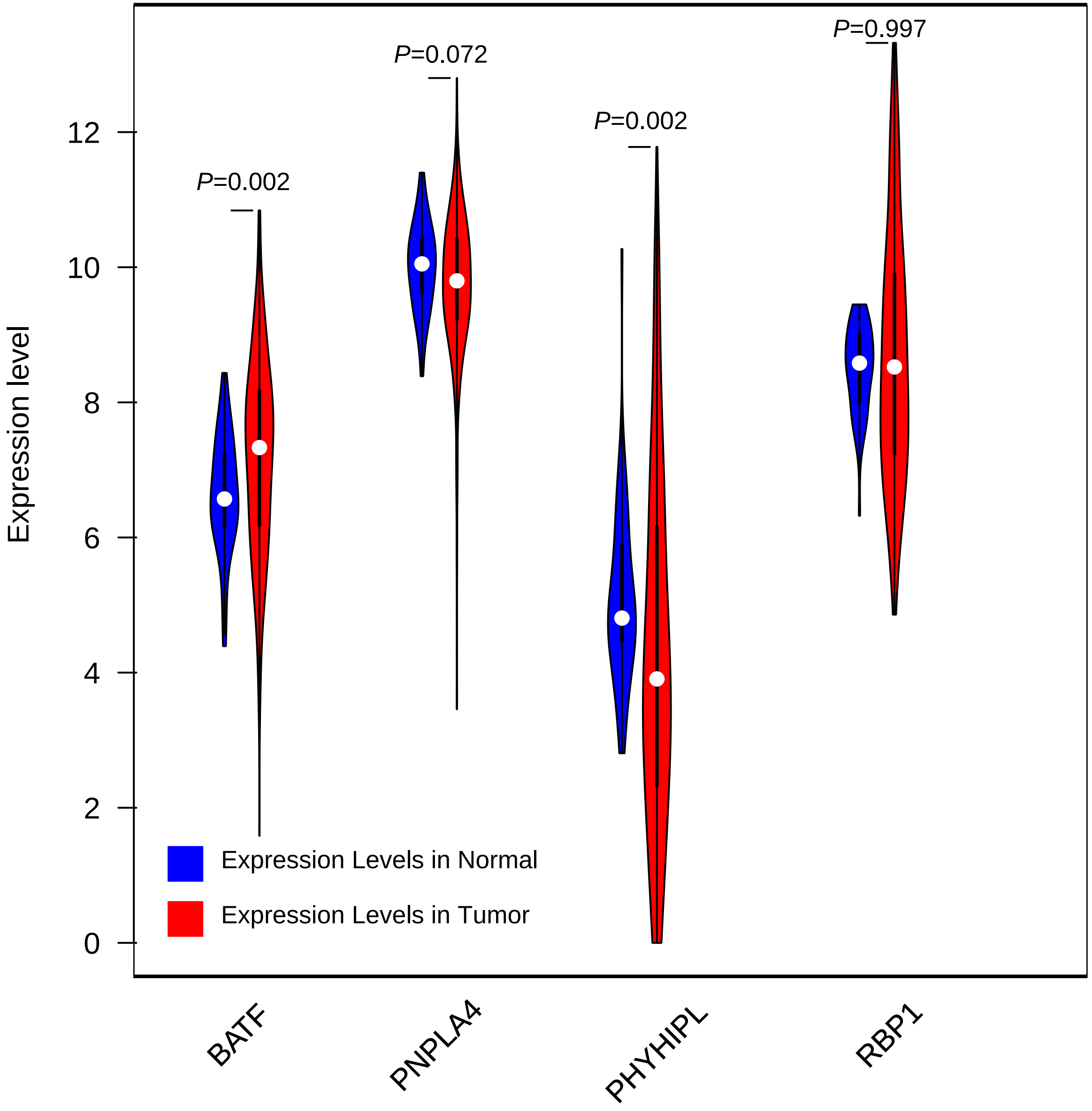

Supplementary Figure S3

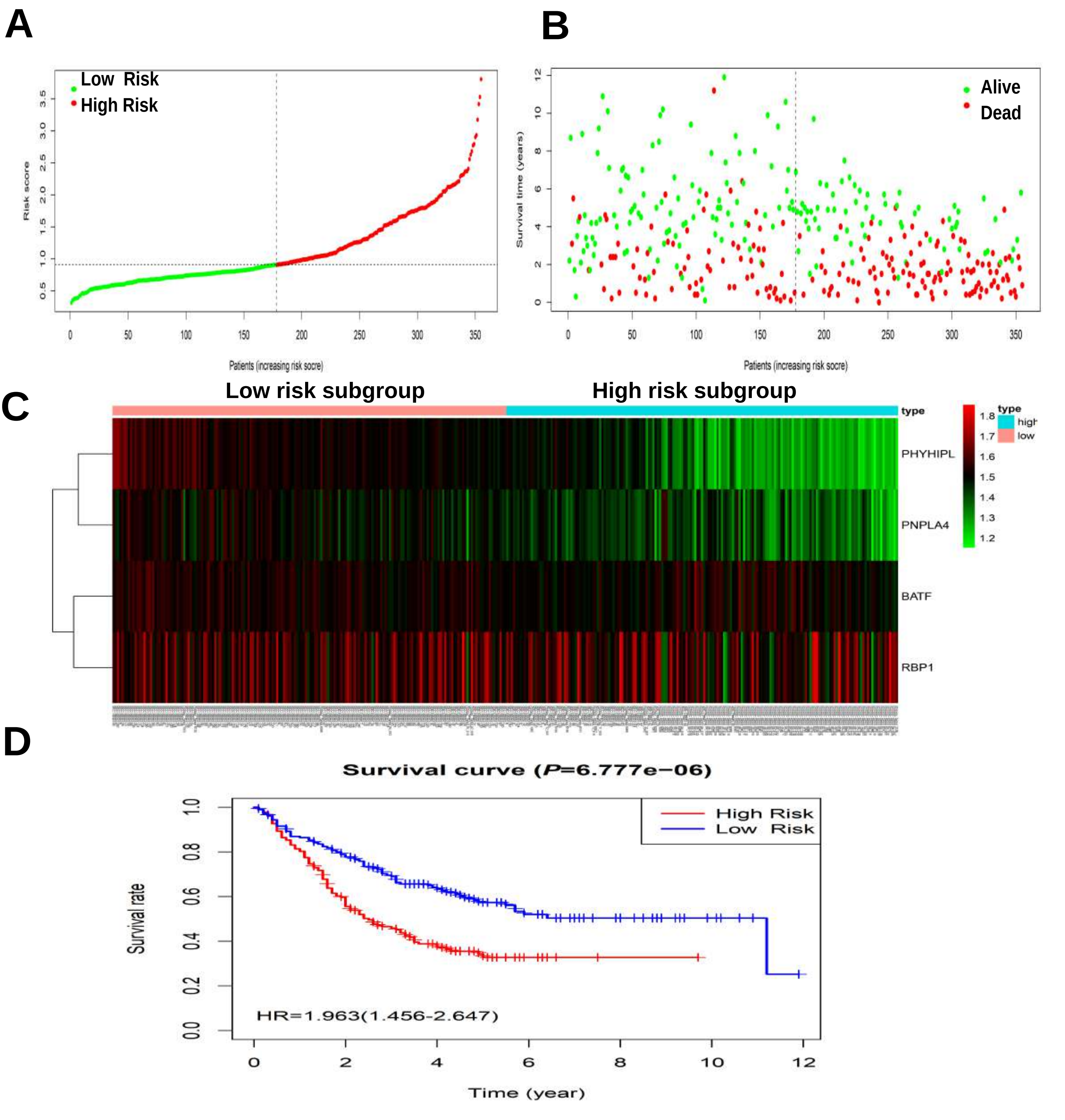

Supplementary Figure S4

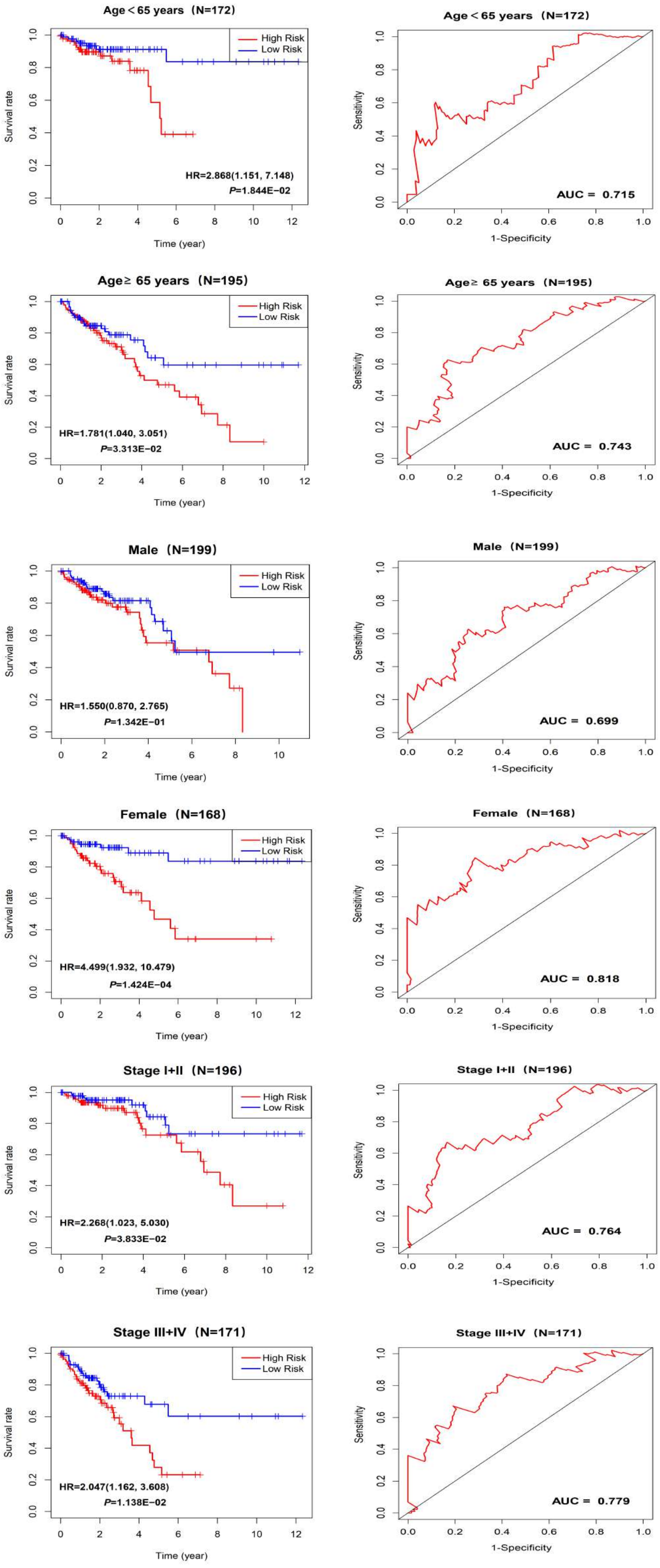

Supplementary Figure S5

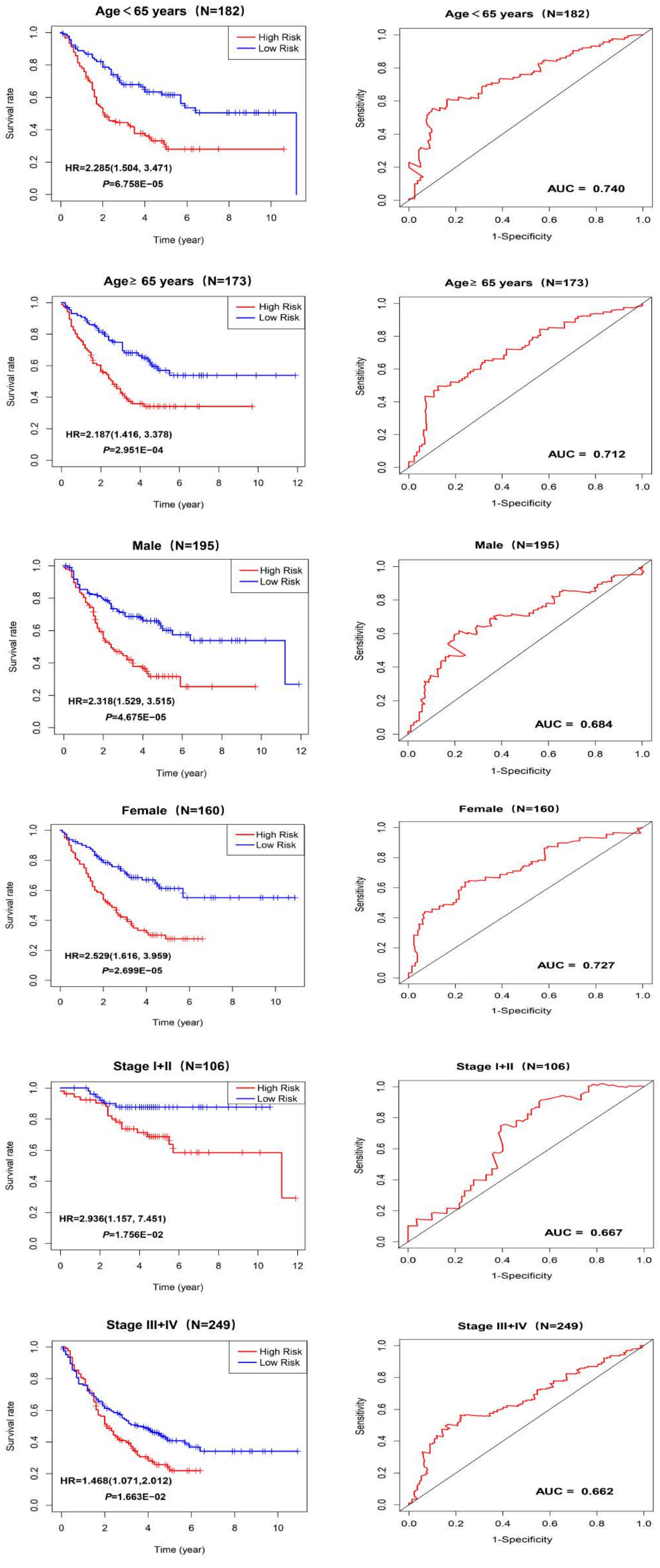

Supplementary Figure S6

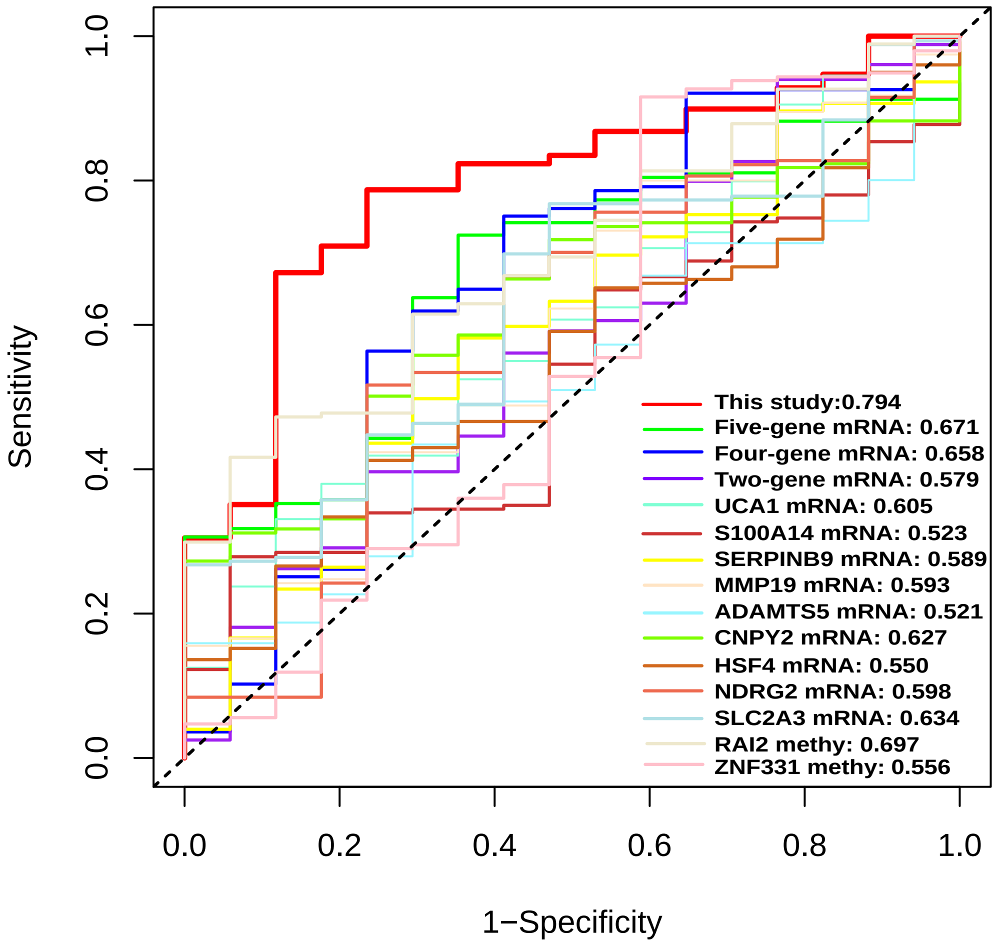

Supplementary Figure S7

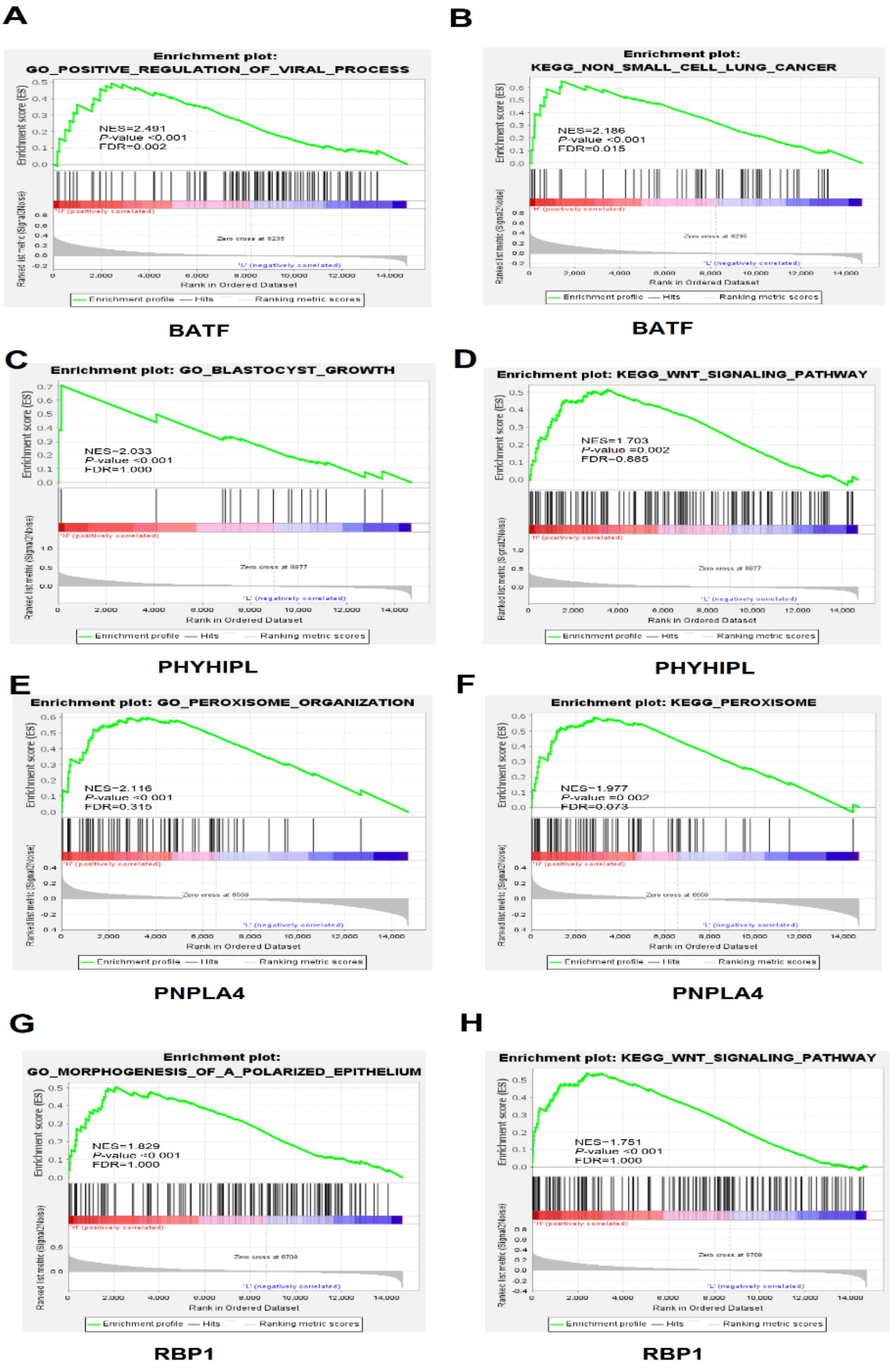

Supplementary Figure S8

II Supplementary Tables

Table S1. 143 epigenetic drivers were identified by MethylMix.

Gene Symbol	NormalMean	TumorMean	logFC	*P*-Value	adjust *P*-Value	cor	cor *P*-value
ABCC2	0.736	0.618	-0.253	0.000	0.000	-0.583	0.000
ADAL	0.059	0.094	0.686	0.000	0.015	-0.559	0.000
AIM2	0.724	0.454	-0.672	0.000	0.000	-0.514	0.000
AKR1B1	0.130	0.473	1.868	0.000	0.000	-0.620	0.000
ANO6	0.083	0.110	0.406	0.000	0.000	-0.508	0.000
ANXA9	0.702	0.530	-0.405	0.000	0.000	-0.561	0.000
AQP5	0.197	0.481	1.292	0.000	0.000	-0.628	0.000
ARHGDIB	0.781	0.550	-0.506	0.000	0.000	-0.519	0.000
B3GALNT1	0.209	0.383	0.874	0.000	0.000	-0.510	0.000
BATF	0.589	0.470	-0.325	0.000	0.000	-0.641	0.000
BST2	0.468	0.298	-0.654	0.000	0.000	-0.670	0.000
C10orf99	0.442	0.654	0.564	0.000	0.000	-0.586	0.000
C3orf14	0.414	0.524	0.340	0.000	0.000	-0.809	0.000
C6orf15	0.807	0.715	-0.174	0.000	0.000	-0.547	0.000
CADPS	0.203	0.178	-0.188	0.000	0.040	-0.576	0.000
CAPN8	0.908	0.571	-0.671	0.000	0.000	-0.505	0.000
CD40	0.226	0.480	1.091	0.000	0.000	-0.502	0.000
CDHR1	0.128	0.190	0.573	0.000	0.000	-0.583	0.000
CDK20	0.141	0.118	-0.248	0.000	0.000	-0.611	0.000
CEACAM6	0.386	0.251	-0.619	0.000	0.000	-0.635	0.000
CEL	0.629	0.504	-0.319	0.000	0.001	-0.795	0.000
CELP	0.747	0.535	-0.480	0.000	0.000	-0.704	0.000
CFTR	0.136	0.099	-0.454	0.000	0.000	-0.519	0.000
CHFR	0.040	0.239	2.560	0.000	0.000	-0.839	0.000
CIDEB	0.497	0.636	0.354	0.000	0.000	-0.517	0.000
DAPP1	0.584	0.416	-0.490	0.000	0.000	-0.704	0.000
DNAJC15	0.787	0.693	-0.183	0.000	0.000	-0.561	0.000
ECHDC3	0.379	0.500	0.398	0.000	0.002	-0.689	0.000
ELAVL2	0.065	0.264	2.013	0.000	0.000	-0.514	0.000
ELF5	0.729	0.602	-0.277	0.000	0.000	-0.789	0.000
ENPP3	0.679	0.768	0.177	0.000	0.000	-0.630	0.000
EREG	0.434	0.332	-0.385	0.000	0.000	-0.776	0.000
FAM131A	0.364	0.391	0.102	0.000	0.000	-0.557	0.000
FAM155B	0.234	0.398	0.766	0.000	0.002	-0.573	0.000
FAM3D	0.470	0.411	-0.195	0.000	0.007	-0.586	0.000
FCGBP	0.761	0.693	-0.134	0.000	0.000	-0.530	0.000
FGF2	0.626	0.739	0.238	0.000	0.000	-0.545	0.000
FLJ32063	0.145	0.327	1.173	0.000	0.000	-0.731	0.000
FUZ	0.046	0.173	1.926	0.000	0.000	-0.651	0.000
FZD10	0.161	0.341	1.080	0.000	0.000	-0.578	0.000
GBGT1	0.513	0.642	0.325	0.000	0.000	-0.576	0.000
GIPC2	0.234	0.184	-0.346	0.000	0.000	-0.532	0.000
GPX2	0.603	0.450	-0.422	0.000	0.000	-0.543	0.000
GPX7	0.272	0.419	0.626	0.000	0.000	-0.607	0.000
GYPC	0.534	0.654	0.293	0.000	0.000	-0.533	0.000
HABP2	0.840	0.670	-0.325	0.000	0.000	-0.601	0.000
HNF4A	0.427	0.323	-0.402	0.000	0.000	-0.530	0.000
HYDIN	0.398	0.566	0.510	0.000	0.000	-0.561	0.000
IFITM1	0.593	0.398	-0.577	0.000	0.000	-0.684	0.000
INPP1	0.562	0.446	-0.334	0.000	0.000	-0.522	0.000
INPP5D	0.587	0.377	-0.639	0.000	0.000	-0.769	0.000
IRAK3	0.372	0.477	0.358	0.000	0.000	-0.583	0.000

IRX2	0.184	0.403	1.130	0.000	0.000	-0.635	0.000
KIF25	0.796	0.634	-0.330	0.000	0.023	-0.613	0.000
KRBA1	0.456	0.702	0.621	0.000	0.000	-0.693	0.000
KRT20	0.517	0.369	-0.485	0.000	0.000	-0.560	0.000
KRT23	0.763	0.546	-0.484	0.000	0.000	-0.559	0.000
KRT7	0.448	0.604	0.430	0.000	0.000	-0.531	0.000
LCMT2	0.059	0.094	0.686	0.000	0.015	-0.522	0.000
LOC286467	0.561	0.326	-0.784	0.000	0.000	-0.549	0.000
LRRC4	0.078	0.444	2.508	0.000	0.000	-0.552	0.000
LY6G6D	0.458	0.345	-0.407	0.000	0.000	-0.728	0.000
MEIS3P1	0.617	0.703	0.188	0.000	0.000	-0.629	0.000
MIA	0.874	0.628	-0.477	0.000	0.000	-0.583	0.000
NINL	0.107	0.302	1.501	0.000	0.005	-0.623	0.000
NMRAL1	0.146	0.121	-0.270	0.000	0.000	-0.549	0.000
NPC1L1	0.758	0.681	-0.154	0.000	0.000	-0.634	0.000
NUDT12	0.297	0.247	-0.266	0.000	0.006	-0.670	0.000
PGC	0.833	0.729	-0.193	0.000	0.000	-0.509	0.000
PHYHIPL	0.095	0.290	1.611	0.000	0.000	-0.531	0.000
PIGR	0.389	0.572	0.555	0.000	0.000	-0.742	0.000
PIPOX	0.447	0.216	-1.047	0.000	0.000	-0.629	0.000
PKIA	0.086	0.380	2.146	0.000	0.000	-0.657	0.000
PNPLA4	0.173	0.167	-0.050	0.000	0.000	-0.508	0.000
PRKY	0.349	0.254	-0.455	0.000	0.015	-0.772	0.000
PTK7	0.249	0.337	0.435	0.000	0.000	-0.590	0.000
QPCT	0.417	0.367	-0.186	0.000	0.000	-0.525	0.000
RAB32	0.212	0.181	-0.228	0.000	0.000	-0.567	0.000
RARRES3	0.681	0.524	-0.379	0.000	0.000	-0.562	0.000
RBP1	0.415	0.536	0.368	0.000	0.000	-0.614	0.000
RNF212	0.590	0.706	0.257	0.000	0.000	-0.570	0.000
RPL39L	0.099	0.383	1.957	0.000	0.000	-0.674	0.000
SERP2	0.321	0.447	0.479	0.000	0.006	-0.818	0.000
SERPINA1	0.528	0.417	-0.340	0.000	0.000	-0.609	0.000
SLC43A3	0.408	0.554	0.440	0.000	0.000	-0.615	0.000
SPAG16	0.144	0.238	0.728	0.000	0.000	-0.768	0.000
SRPX2	0.682	0.505	-0.434	0.000	0.000	-0.698	0.000
SULT4A1	0.291	0.506	0.799	0.000	0.000	-0.531	0.000
TBX18	0.172	0.475	1.466	0.000	0.000	-0.738	0.000
TMEM106A	0.078	0.302	1.959	0.000	0.028	-0.700	0.000
TMEM173	0.235	0.205	-0.201	0.000	0.036	-0.644	0.000
TMEM220	0.442	0.636	0.524	0.000	0.000	-0.589	0.000
TMEM71	0.728	0.617	-0.239	0.000	0.019	-0.603	0.000
VSIG2	0.550	0.684	0.315	0.000	0.000	-0.602	0.000
WDR35	0.147	0.131	-0.167	0.000	0.000	-0.608	0.000
ZFP28	0.070	0.403	2.524	0.000	0.000	-0.746	0.000
ZFP82	0.140	0.448	1.681	0.000	0.000	-0.714	0.000
ZIK1	0.400	0.621	0.635	0.000	0.000	-0.536	0.000
ZNF134	0.044	0.281	2.681	0.000	0.000	-0.752	0.000
ZNF141	0.086	0.175	1.020	0.000	0.002	-0.733	0.000
ZNF256	0.049	0.274	2.471	0.000	0.000	-0.789	0.000
ZNF264	0.393	0.477	0.279	0.000	0.000	-0.622	0.000
ZNF285	0.119	0.354	1.574	0.000	0.000	-0.790	0.000
ZNF287	0.083	0.214	1.361	0.000	0.000	-0.616	0.000
ZNF300	0.343	0.506	0.559	0.000	0.000	-0.795	0.000
ZNF304	0.108	0.405	1.902	0.000	0.000	-0.743	0.000
ZNF331	0.378	0.556	0.556	0.000	0.000	-0.616	0.000
ZNF334	0.304	0.606	0.995	0.000	0.000	-0.541	0.000

ZNF347	0.164	0.381	1.215	0.000	0.000	-0.701	0.000
ZNF354C	0.079	0.323	2.024	0.000	0.000	-0.611	0.000
ZNF415	0.274	0.500	0.868	0.000	0.000	-0.680	0.000
ZNF43	0.147	0.431	1.549	0.000	0.000	-0.778	0.000
ZNF461	0.136	0.294	1.108	0.000	0.000	-0.623	0.000
ZNF471	0.155	0.457	1.560	0.000	0.000	-0.630	0.000
ZNF480	0.057	0.113	0.982	0.000	0.009	-0.734	0.000
ZNF506	0.263	0.305	0.215	0.000	0.002	-0.547	0.000
ZNF525	0.157	0.265	0.752	0.000	0.000	-0.666	0.000
ZNF528	0.116	0.412	1.829	0.000	0.000	-0.713	0.000
ZNF529	0.217	0.390	0.847	0.000	0.000	-0.641	0.000
ZNF543	0.047	0.320	2.776	0.000	0.000	-0.770	0.000
ZNF547	0.040	0.128	1.675	0.000	0.000	-0.657	0.000
ZNF549	0.264	0.513	0.957	0.000	0.000	-0.807	0.000
ZNF568	0.127	0.458	1.856	0.000	0.000	-0.551	0.000
ZNF569	0.201	0.387	0.942	0.000	0.000	-0.664	0.000
ZNF570	0.172	0.364	1.083	0.000	0.000	-0.747	0.000
ZNF582	0.188	0.517	1.458	0.000	0.000	-0.600	0.000
ZNF583	0.063	0.150	1.244	0.000	0.005	-0.627	0.000
ZNF625	0.044	0.443	3.329	0.000	0.000	-0.563	0.000
ZNF655	0.042	0.229	2.435	0.000	0.000	-0.815	0.000
ZNF662	0.423	0.636	0.588	0.000	0.000	-0.621	0.000
ZNF677	0.381	0.615	0.691	0.000	0.000	-0.501	0.000
ZNF682	0.056	0.236	2.066	0.000	0.000	-0.768	0.000
ZNF75A	0.245	0.513	1.067	0.000	0.000	-0.550	0.000
ZNF772	0.266	0.451	0.763	0.000	0.000	-0.618	0.000
ZNF793	0.043	0.424	3.290	0.000	0.000	-0.737	0.000
ZNF813	0.093	0.274	1.551	0.000	0.000	-0.711	0.000
ZNF829	0.127	0.458	1.856	0.000	0.000	-0.555	0.000
ZNF83	0.137	0.258	0.916	0.000	0.001	-0.565	0.000
ZNF844	0.071	0.141	0.986	0.000	0.002	-0.604	0.000
ZNF85	0.285	0.537	0.917	0.000	0.000	-0.599	0.000
ZNF880	0.125	0.419	1.741	0.000	0.000	-0.576	0.000
ZSCAN18	0.373	0.502	0.429	0.000	0.000	-0.660	0.000
ZSCAN23	0.144	0.477	1.729	0.000	0.000	-0.579	0.000

Table S2. Identificaion of six methylation-driven genes by multivariable Cox regression analysis.

Gene symbol	Coefficient[a]	HR	HR(95%Low)	HR(95%High)	*P* -value[a]
ANXA9	0.261	1.298	1.038	1.623	0.022
BATF	0.247	1.281	1.084	1.513	0.004
PHYHIPL	0.136	1.146	1.035	1.268	0.009
PNPLA4	-0.224	0.799	0.658	0.970	0.023
RBP1	-0.232	0.793	0.688	0.914	0.001
SERPINA1	-0.195	0.823	0.713	0.950	0.008

[a]Derived from the multivariable Cox regression analysis in the training set.

Table S3. Compared the overall survival prediction and AUC values of 63 different combined models in the training set.

Combined Model		Overall Survival				AUC[a]
		HR	HR(95%Low)	HR(95%High)	*P*-value	
One Gene Model	ANXA9	1.420	0.929	2.173	0.104	0.569 (5)
	BATF	1.763	1.144	2.717	0.009	0.701
	PHYHIPL	1.358	0.890	2.070	0.154	0.677 (6)
	PNPLA4	1.466	0.960	2.239	0.075	0.731
	RBP1	1.103	0.726	1.676	0.647	0.652
	SERPINA1	1.730	1.128	2.653	0.011	0.608
Two Genes Model	A-B	2.184	1.404	3.398	0.000	0.645
	A-P1	1.583	1.032	2.428	0.034	0.636 (6)
	A-P2	1.845	1.197	2.843	0.005	0.660
	A-R	1.628	1.065	2.487	0.023	0.608 (5)
	A-S	1.860	1.207	2.865	0.004	0.567
	B-P1	2.116	1.367	3.277	0.001	0.699
	B-P2	1.883	1.222	2.901	0.004	0.772
	B-R	1.728	1.126	2.653	0.011	0.811
	B-S	2.305	1.475	3.600	0.000	0.724
	P1-R	1.563	1.025	2.384	0.004	0.671 (6)
	P1-S	1.637	1.069	2.506	0.022	0.649 (6)
	P2-S	1.966	1.276	3.028	0.002	0.705
	P2-R	1.592	1.043	2.430	0.030	0.752
	P1-P2	1.587	1.036	2.430	0.032	0.710
	R-S	1.716	1.121	2.627	0.012	0.689
Three Genes Model	A-B-P1	1.991	1.288	3.076	0.002	0.673 (6)
	A-B-P2	2.527	1.598	3.997	0.000	0.742
	A-B-R	2.227	1.437	3.451	0.000	0.704
	A-B-S	2.414	1.539	3.785	0.000	0.691
	A-P1-P2	1.827	1.181	2.826	0.006	0.664
	A-P1-R	1.973	1.285	3.031	0.002	0.683 (5)
	A-P1-S	1.687	1.100	2.589	0.015	0.645
	A-P2-R	1.940	1.261	2.982	0.002	0.670
	A-P2-S	2.120	1.364	3.295	0.001	0.666
	A-R-S	2.015	1.309	3.101	0.001	0.625
	B-P1-P2	1.872	1.212	2.891	0.004	0.803
	B-P1-R	1.692	1.101	2.601	0.015	0.832
	B-P1-S	2.445	1.562	3.826	0.000	0.728
	B-P2-R	1.723	1.122	2.648	0.012	0.823
	B-P2-S	2.559	1.625	4.031	0.000	0.776
	B-R-S	2.445	1.564	3.823	0.000	0.793
	P1-P2-R	2.037	1.323	3.139	0.001	0.749 (7)
	P1-P2-S	1.977	1.279	3.056	0.002	0.698
	P1-R-S	1.928	1.255	2.962	0.002	0.705
	P2-R-S	1.629	1.065	2.493	0.023	0.746
Four Genes Model	P1-P2-R-S	1.902	1.240	2.918	0.003	0.759
	B-P2-R-S	2.739	1.736	4.320	0.000	0.822
	B-P1-R-S	2.301	1.477	3.583	0.000	0.808
	B-P1-P2-S	2.459	1.567	3.860	0.000	0.780
	B-P1-P2-R	2.184	1.404	3.396	0.000	0.876
	A-P2-R-S	2.299	1.483	3.565	0.000	0.693
	A-P1-R-S	2.285	1.477	3.534	0.000	0.660
	A-P1-P2-S	1.926	1.247	2.977	0.003	0.669
	A-P1-P2-R	2.125	1.378	3.275	0.000	0.707
	A-B-P2-S	2.443	1.552	3.845	0.000	0.749
	A-B-P2-R	2.278	1.468	3.537	0.000	0.763

	A-B-P1-S	2.296	1.477	3.569	0.000	0.703
	A-B-P1-R	2.365	1.514	3.693	0.000	0.745
	A-B-R-S	2.617	1.673	4.093	0.000	0.735
	A-B-P1-P2	2.703	1.707	4.279	0.000	0.754
Five Genes Model	B-P1-P2-R-S	2.227	1.435	3.454	0.000	0.840
	A-P1-P2-R-S	1.819	1.186	2.790	0.005	0.724
	A-B-P2-R-S	3.240	2.035	5.160	0.000	0.778
	A-B-P1-R-S	2.809	1.779	4.435	0.000	0.761
	A-B-P1-P2-S	2.533	1.608	3.989	0.000	0.762
	A-B-P1-P2-R	2.573	1.645	4.023	0.000	0.804
Six Genes Model	A-B-P1-P2-R-S	2.648	1.688	4.155	0.000	0.796

Note:"A" means ANXA9, "B" means BATF, "P1" means PHYHIPL, "P2" means PNPLA4, "R" means RBP1, "S" mea SEPRINA1. "(5)" means predicting 5-year overall survival of patients

[a]=AUC based on the ROC curves for the sensitivity and specificity of different signatures in predicting 9-year overall survival of patients.

Table S4. The C-index of TNM staging system and nomograms.

Models	Training set (N=367)		Testing set (N=355)	
	C-index (95%CI)	*P* -value	C-index (95%CI)	*P* -value
TNM staging	0.719 (0.654-0.783)	<0.001	0.706 (0.669-0.743)	<0.001
Nomogram	0.747 (0.687-0.807)	<0.001	0.707 (0.670-0.743)	<0.001

Table S5. Compared the prognostic effect of four methylation-driven genes signature with other partially known biomarkers.

Signature	AUC	95% CI of AUC	Type	*P* value[a]	Ref[b]
This study	0.794	0.681-0.907	Protein coding		This study
Five-gene mRNA	0.671	0.529-0.813	Protein coding	0.182	[1]
Four-gene mRNA	0.658	0.501-0.816	Protein coding	0.211	[2]
Two-gene mRNA	0.579	0.423-0.735	Protein coding	0.040	[3]
UCA1	0.605	0.456-0.754	Protein coding	0.030	[4]
S100A14	0.523	0.382-0.660	Protein coding	0.004	[5]
SERPINB9	0.589	0.437-0.742	Protein coding	0.009	[6]
MMP19	0.593	0.440-0.746	Protein coding	0.025	[7]
ADAMTS5	0.521	0.375-0.668	Protein coding	0.001	[8]
CNPY2	0.627	0.495-0.760	Protein coding	0.064	[9]
HSF4	0.550	0.403-0.697	Protein coding	0.008	[10]
NDRG2	0.598	0.437-0.758	Protein coding	0.028	[11]
SLC2A3	0.634	0.495-0.773	Protein coding	0.087	[12]
RAI2	0.697	0.571-0.823	Methylation	0.223	[13]
ZNF331	0.556	0.377-0.734	Methylation	0.018	[14]

[a]:in the statistical comparison (Z test) between AUC value of corresponding signature and " this study" signature.

[b]:Additional References:

[1]. Sun G, Li Y, Peng Y, et al. Identification of a five-gene signature with prognostic value in colorectal cancer. Journal of cellular physiology. 2019;234(4):3829-36.

[2]. Li X, Zhang Q, Zhao L, et al. A Combined four-mRNA Signature Associated with Lymphatic Metastasis for Prognosis of Colorectal Cancer. Journal of Cancer. 2020;11(8):2139-49.

[3]. Abdel Raouf SM, Ibrahim TR, Abdelaziz LA, et al. Prognostic Value of TWIST1 and EZH2 Expression in Colon Cancer. Journal of gastrointestinal cancer. 2019.

[4]. Liu X, Liu X, Qiao T, et al. Prognostic and clinicopathological significance of long non-coding RNA UCA1 in colorectal cancer: Results from a meta-analysis. Medicine. 2019;98(48):e18031.

[5]. Diamantopoulou A, Mantas D, Kostakis ID, et al. A Clinicopathological Analysis of S100A14 Expression in Colorectal Cancer. In vivo (Athens, Greece). 2020;34(1):321-30.

[6]. Vycital O, Pitule P, Hosek P, et al. Expression of Serpin B9 as a Prognostic Factor of Colorectal Cancer. Anticancer research. 2019;39(11):6063-6.

[7]. Chen Z, Wu G, Ye F, et al. High expression of MMP19 is associated with poor prognosis in patients with colorectal cancer. BMC cancer. 2019;19(1):448.

[8]. Li J, Liao Y, Huang J, et al. Epigenetic silencing of ADAMTS5 is associated with increased invasiveness and poor survival in patients with colorectal cancer. Journal of cancer research and clinical oncology. 2018;144(2):215-27.

[9]. Peng J, Ou Q, Pan Z, Zhang R, Zhao Y, Deng Y, et al. Serum CNPY2 isoform 2 represents a novel biomarker for early detection of colorectal cancer. Aging. 2018;10(8):1921-31.

[10].Yang Y, Jin L, Zhang J, et al. High HSF4 expression is an independent indicator of poor overall survival and recurrence free survival in patients with primary colorectal cancer. IUBMB life. 2017;69(12):956-61.

[11].Chen W, Peng J, Ou Q, et al. Expression of NDRG2 in Human Colorectal Cancer and its Association with Prognosis. Journal of Cancer. 2019;10(15):3373-80.

[12].Kim E, Jung S, Park WS, et al. Upregulation of SLC2A3 gene and prognosis in colorectal carcinoma: analysis of TCGA data. BMC cancer. 2019;19(1):302.

[13].Yan W, Wu K, Herman JG, et al. Retinoic acid-induced 2 (RAI2) is a novel tumor suppressor, and promoter region methylation of RAI2 is a poor prognostic marker in colorectal cancer. Clinical epigenetics. 2018;10:69.

[14].Wang Y, He T, Herman JG, et al. Methylation of ZNF331 is an independent prognostic marker of colorectal cancer and promotes colorectal cancer growth. Clinical epigenetics. 2017;9:115.
